# A Mouse Model of Pulmonary Metastasis from Spontaneous Osteosarcoma Monitored *In Vivo* by Luciferase Imaging

**DOI:** 10.1371/journal.pone.0001828

**Published:** 2008-03-19

**Authors:** Silvia Miretti, Ilaria Roato, Riccardo Taulli, Carola Ponzetto, Michele Cilli, Martina Olivero, Maria Flavia Di Renzo, Laura Godio, Adriana Albini, Paolo Buracco, Riccardo Ferracini

**Affiliations:** 1 Department of Morphophysiology, School of Veterinary Medicine, University of Torino, Grugliasco, Turin, Italy; 2 Center for Experimental Research and Medical Studies (CeRMS) University and A.S.O. San Giovanni Battista, Turin, Italy; 3 Department of Anatomy, Pharmacology and Forensic Medicine, University of Torino, Turin, Italy; 4 Animal Research Facility, National Institute for Cancer Research, Genoa, Italy; 5 Laboratory of Cancer Genetics of the Institute for Cancer Research and Treatment (IRCC), University of Torino School of Medicine, Candiolo, Turin, Italy; 6 Laboratory of Pathology, Department of Pathology, A.S.O. San Giovanni Battista, Turin, Italy; 7 IRCCS Multimedica Science and Technology Park, Milano, Italy; 8 Department of Animal Pathology, School of Veterinary Medicine, University of Torino, Grugliasco, Turin, Italy; 9 Department of Orthopaedics, A.S.O. San Giovanni Battista, Turin, Italy; Katholieke Universiteit Leuven, Belgium

## Abstract

**Background:**

Osteosarcoma (OSA) is lethal when metastatic after chemotherapy and/or surgical treatment. Thus animal models are necessary to study the OSA metastatic spread and to validate novel therapies able to control the systemic disease. We report the development of a syngeneic (Balb/c) murine OSA model, using a cell line derived from a spontaneous murine tumor.

**Methodology:**

The tumorigenic and metastatic ability of OSA cell lines were assayed after orthotopic injection in mice distal femur. Expression profiling was carried out to characterize the parental and metastatic cell lines. Cells from metastases were propagated and engineered to express Luciferase, in order to follow metastases *in vivo*.

**Principal Findings:**

Luciferase bioluminescence allowed to monitor the primary tumor growth and revealed the appearance of spontaneous pulmonary metastases. *In vivo* assays showed that metastasis is a stable property of metastatic OSA cell lines after both propagation in culture and luciferase trasduction. When compared to parental cell line, both unmodified and genetically marked metastatic cells, showed comparable and stable differential expression of the *enpp4*, *pfn2* and *prkcd* genes, already associated to the metastatic phenotype in human cancer.

**Conclusions:**

This OSA animal model faithfully recapitulates some of the most important features of the human malignancy, such as lung metastatization. Moreover, the non-invasive imaging allows monitoring the tumor progression in living mice. A great asset of this model is the metastatic phenotype, which is a stable property, not modifiable after genetic manipulation.

## Introduction

Osteosarcoma (OSA) is the most common primary malignant tumor of bone. OSA derives from primitive bone-forming mesenchyme and it is characterized by the proliferating spindle cells production of osteoid tissue or immature bone. OSA accounts for 60% of all malignant childhood bone tumors [1]. The age distribution is bimodal: the first major peak occurring during the second decade of life, and the second much smaller peak being observed in patients over 50 years-old. The distal femoral and proximal tibial metaphyses are the most common sites for OSA. Approximately 50% of cases are localized in the knee region [2]. OSA is a progressive and often fatal disease [3]. In absence of adjuvant therapy, removal and treatment of the primary tumor is successful in only 20% of the cases, while chemotherapy regiments combined to surgery reduces relapses and metastases under 40%. At diagnosis, approximately 80% of patients are believed to have metastatic disease, but the current diagnostic tools allow to detect only 8–15% of these patients [4]. In particular, many patients bear undetectable pulmonary micrometastases. In fact, a distinct feature of OSA is a preference for pulmonary metastases compared with other sites. After radiological detection of lung metastasis, 5 years survival rates are less than 30% [5]. Thus new therapies are necessary, and the development of clinically relevant animal models could be really helpful for this purpose.

The mouse model provides the most relevant setting to study and molecularly dissect the process of metastasis *in vivo*. It is interesting to note that metastatic spread of experimental or spontaneous tumors is a surprisingly rare event in murine compared to human cancer. For this reason few mouse models are available to study the metastatic phenotype and its progression [6]. Several experimental assays have been developed in mice to assess the metastatic ability of OSA cells, which can be injected in specific anatomical sites, analyzing their ability to form tumors and/or to metastasize via haematogenous spread [7]. Recently, the development of a novel bioluminescent *in vivo* imaging system (IVIS) allowed monitoring in real-time labeled substances or cells in experimental animals. In cancer research this technology was successfully used to study tumor progression and to verify efficacy of new therapies [8], [9]. IVIS has been also used to monitor viral [10] and bacterial infections [11], [12], to study transcriptional regulation of human genes [13] and to evaluate the distribution of drugs [14].

In this work we describe the establishment of a Luciferase-labelled metastatic OSA model, which can be monitored *in vivo*. Highly and poorly metastatic cell lines were originally established from an OSA, spontaneously arising in a Balb/c mouse [15], [16]. Metastatic cells were selected *in vivo* and engineered to express a reporter gene (Luciferase). Upon orthotopic injection, i.e. into the right distal femur of syngeneic hosts, IVIS allowed monitoring tumor growth and metastases. IVIS assay and gene expression showed that the metastatic OSA cells phenotype was maintained after propagation in culture and gene marking.

## Materials and Methods

### OSA cell culture and characterization

Different OSA cell lines (K1–K5) were provided by Dr. J. Schmidt [15]. Lines had been obtained from the primary culture of a spontaneous OSA which arose in the distal femur of an 894-day-old female Balb/c mouse [15]. Cells were grown in DMEM (Dulbecco Modified Eagle Medium, Cambrex, Walkersville, MD) supplemented with 10% FBS (Fetal Bovine Serum, Cambrex, Walkersville, MD), 2 mM L-Glutamine (Cambrex, Walkersville, MD), 10000 U.I./mL penicillin-streptomycin (Cambrex, Walkersville, MD) and incubated at 37°C in a 5% CO_2_ water saturated atmosphere K5L cells were obtained by a K5 metastatic lung nodule excision, generated from K5 cell line orthotopic injection.

OSA cells were tested for alkaline phosphatase (ALP) expression by immunohistochemistry. 3×10^5^ cells were plated in a 24 multiwell plate, after adhesion they were stained and ALP expression was quantified using the method of scoring described in the manifacturer's instructions (Sigma Aldrich, St. Louis, MO). Briefly, we evaluated the intensity of staining using fibroblast as negative control, while our cells showed a strong intensity of stain.

### Total RNA extraction and Real-time PCR

Total RNA was prepared as previously described [17]. To determine the amount of specific RNA products of the ecto-nucleotide pyrophosphatase/phosphodiesterase (*enpp4*), profilin2 (*pfn2*) and Protein Kinase Cδ (*prkcd*) genes, cDNA was subjected to quantitative PCR, using Sybr green Master MIX (Applied Biosystem, Foster City, CA) and MyiQ single-color Real Time PCR detection System (Biorad Inc., Hercules, CA). Each experiment was repeated three times independently, to ensure the reproducibility of results. Primer sequences were designed using Primer Express Software (Version 1.5). Sequences are available from the authors.

### Motility and Matrigel® invasion assay

Motility and invasion assays were performed in 6.5 mm diameter transwell chambers (Costar, Cambridge, MA). The upper side of the porous polycarbonate membrane (8.0 µm pore size) was coated with 10 µg/cm^2^ reconstituted Matrigel® basement membrane (Collaborative Biomedical Products; Becton Dickinson Labware, Waltham, MA). Cells (2.5×10^4^/well) were seeded on the upper side of the filter and incubated in DMEM medium and 0,5% FBS. The lower chamber was filled with medium containing 0,5% FBS.

After 16h for motility assay or 24h for invasion assay, cells on the upper side of the filters were mechanically removed. Cells migrated to the lower side were fixed with 11% glutaraldehyde in Phosphate Buffer Saline (PBS) and stained with 0,1% crystal violet in 20% methanol. The filters were photographed and cells were semi-quantitatively counted. MDCK cell line was used as motility and invasiveness control in presence of Hepatocyte Growth Factor (100 ng/ml).

### Anchorage-independent cell-growth assay

Cells were suspended in 0,35% type VII low melting agarose in DMEM (10% FBS) at 2×10^4^ cells/well, plated on a layer of 0,7% agarose in DMEM (10% FBS) in 6-well culture plates and cultured at 37°C with 5% CO_2_. After three weeks colonies >100 µm in diameter were counted.

### Vector construction

Lenti-LUC was generated by subcloning of *firefly*-luciferase gene from pGL3-Control (Promega, Madison, WI) in SmaI and XbaI sites of pCCL.sin.cPPT.polyA.CTE.eGFP.minhCMV.hPGK.deltaNGFR.WPRE transfer vector, which represents a 3rd generation of lentiviral vector (self-inactivating lentiviral vector) [18]. For the amplification of plasmid, the ligation product was used to transform TOP TEN Bacteria (Invitrogen, Carlsbad, CA). These bacteria were selected on ampicillin-agar plate at 37°C for 16 h, then bacteria with the interest plasmid were grown in 200 mL of LB medium (Bacto-tryptone 10 g, bacto-yeast extract 5 g, NaCl 10 g) with ampicillin. The plasmid DNA was extracted with GenElute™ High Performance Plasmid Maxiprep Kit (Sigma Aldrich, St. Louis, MO).

### Lentiviral vector production

High titer lentiviral vector stock was produced in 293T cells by calcium phosphate-mediated transient transfection of pCCL.sin.cPPT.polyA.CTE.eGFP.minhCMV.hPGK.Luciferase.Wprep (10 µg) and packaging vectors pMDLg/pRRE (5 µg), pRSV-REV (2.5 µg) and pMD2.VSVG (5 µg). Supernatants were harvested after 30 h, filtered through 0.22 µm pore size filters (Millipore, Billerica, MA) and used directly or after concentration by ultracentrifugation (50,000×g for 2 h). K5L cells were plating 12 h before (1×10^5^ in 35 mm diameter culture dishes) and were transduced by the virus described above obtaining K5L-Luc. The medium was changed 24h after infection.

### Luciferase assay

The luciferase activity of K5L-Luc cells was tested by the Luciferase Assay System purchased from Promega (Madison, WI). As negative control we used both K5 and K5L cells. Briefly, 4×10^5^ cells were treated with lysis solution and 50 µl of cell suspension was added to 100 µl of luciferin mix. The luciferase activity was quickly measured by a luminometer with 10 seconds integration time.

### Animal model

Female 4–5 week old Balb/c mice (purchased by Charles River Laboratories, Calco, Milano) were housed under pathogen free conditions with a 12 h light/12 h dark schedule, fed autoclaved standard chow and water *ad libitum*.

Mice were manipulated and housed according to protocols approved by the Turin University Bioethical Committee and the Italian Ministry of Health.

For the intra-femur injections, exponentially growing K5, K5L and K5-Luc OSA cells were harvested, counted and resuspended in PBS to a final concentration of 10^7^ cells/ml. The animals were anesthetized with ketamine (80 mg/kg) and xylazine (7 mg/kg). The knee of the Balb/c mouse was fixed beyond 90° and 1×10^6^ cells resuspended in 100 µl of PBS was injected into the distal femur using a 25 gauge needle.

Weekly, mice were monitored for tumor size and evidence of morbidity related to the primary tumor or pulmonary metastases.

Tumor size was quantified in two dimensions using calipers. Tumor volume was calculated as follows: tumor volume (mm^3^) = D×d^2^/2, where D represents the largest cross sectional diameter (mm) of the tumor and d the cross sectional diameter (mm) at right angles to D.

### *In Vivo* Imaging System (IVIS)

IVIS consists of a highly sensitive, charge-coupled digital camera with accompanying advanced computer software for image data acquisition and analysis. This system captures photons of light emitted by reagents or cells that have been coupled or engineered to produce bioluminescence in the living animal [19]. The substrate luciferin was injected into the intraperitoneal cavity at a dose of 150 mg/kg body weight (30 mg/ml luciferin), approximately 5 minutes before imaging. Mice were anesthetized with isoflurane/oxygen and placed on the imaging stage. Ventral and dorsal images were collected for 30 seconds to 2 minutes using the IVIS (Xenogen Corp., Alameda, CA). Photons emitted from the primary tumor and lung region were quantified using Living Image software (Xenogen Corp., Alameda, CA).

### India ink assay

Pulmonary metastases were enumerated by intra-tracheal injection of india ink (15% India Ink, 85% water, 3 drops NH4OH/100 ml). India ink injected lungs were washed in Feket's solution (300 ml 70% EtOH, 30 ml 37% formaldehyde, 5 ml glacial acetic acid) and then placed fresh Feket's solution overnight. White tumor nodules against a blue lung background were counted.

### Histopathological analysis

The primary tumors and the lungs were harvested at necropsy and fixed in 10% formalin. The bone specimens were subjected to Cal-Ex decalcifying solution overnight. The fixed samples were then embedded in paraffin and three non-sequential serial sections per animal were obtained. The sections were stained with hematoxylin/eosin and analyzed for the presence of metastases by light microscopy. The total number of metastases per lung section was counted and averaged among the animals.

### Statistical analyses

Statistical analyses were performed with the Statistical Package for the Social Sciences (spssx/pc) software (SPSS, Chicago, IL, USA). Continuous variables were expressed as mean and standard deviation. Univariate analyses were performed by one way analysis of variance. The results were considered statistically significant for *p*<0.05.

## Results

### Characterization of the murine OSA cell lines

**Figure 1:**
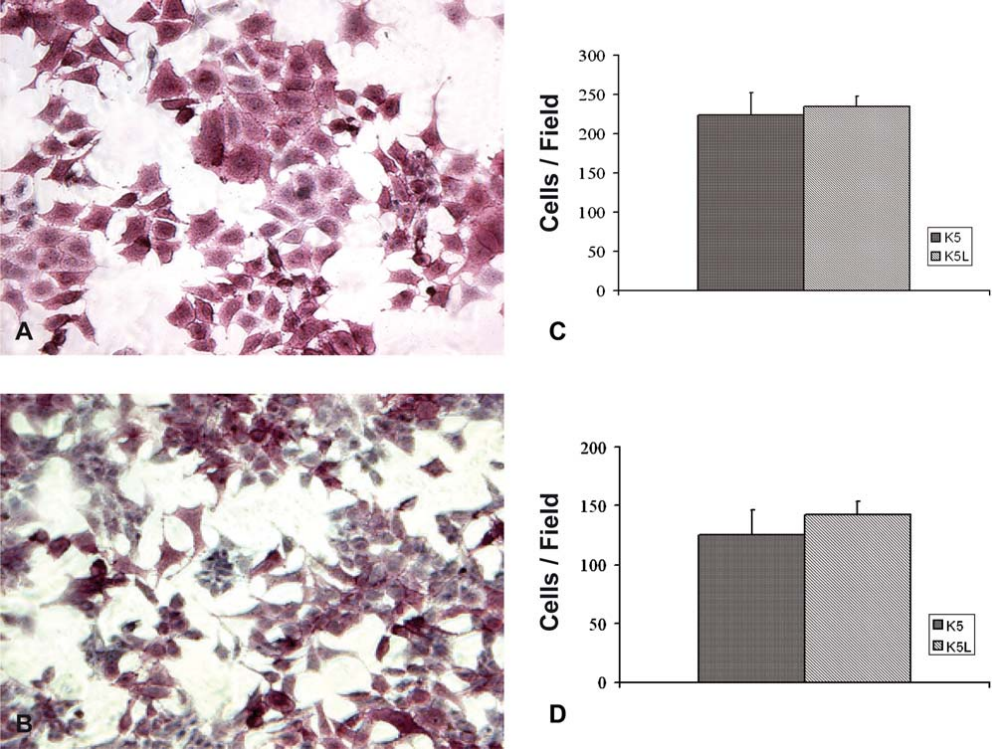
* In vitro* characterization of OSA cell lines. ALP expression in K5 (A) and K5L (B) cells was detected using cytochemical method and analyzed by light microscopy. Both cell lines showed comparable protein expression. (Magnification 20×). To assess the biological features of K5 and K5L cells, cell motility (C) and invasiveness (D) were evaluated in transwell chambers in starving condition. Cells migrated through the porous filter were counted after 16 h incubation (C). Cells migrated through the artificial basement membrane (Matrigel®) were counted after 24 h incubation (D). In both panels Y-axis shows average migrated cell number (±S.D.) of triplicate wells in a representative experiment.

Continuous cell lines (K1–K5) were obtained from a spontaneous OSA, which arose in the distal femur of a Balb/c mouse [15]. We injected orthotopically a metastatic cell line (K5), i.e. in the distal femur of syngeneic mice, and obtained some metastases. One of these metastases was propagated *in vitro* (K5L) and further analyzed. Propagated K5L cells were re-injected orthotopically and found to be more tumorigenic than parental K5 cells. In fact, after 3 weeks, 10/10 of injected mice showed tumor growth, while tumor grew in only 6/10 animals which received the K5 parental cell line. Since ALP expression is a marker of the osteoblast lineage [20], we compared ALP protein expression between K5 and K5L cell lines. The assay indicated strong ALP positivity in both cell lines (Fig. 1 A–B).

We investigated cell motility and invasion ability of the parental K5 and the metastasis-derived K5L cells. We used an artificial matrix containing laminin and Collagen I (the predominant matrix molecules in bone) and we showed that the parental and the metastatic cells had similar ability to migrate across the membrane (Fig 1 C–D). An anchorage-independent growth is a sign of increased aggressiveness, thus we cultured cells in soft agar showing that both the K5 and the K5L cells formed colonies in a soft agar suspension within 17 days (data not shown).

**Figure 2:**
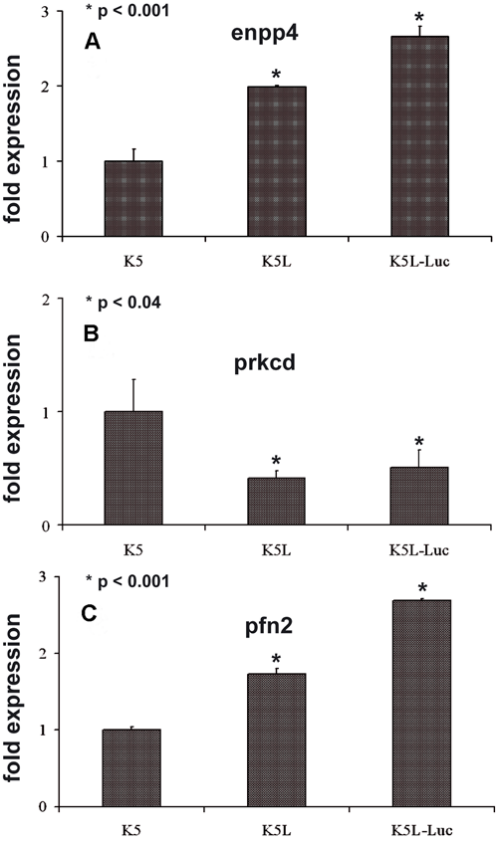
Expression of the *enpp4, prkcd*, *pfn2* genes in OSA cell lines. The amount of *enpp4, prkcd*, *pfn2* transcript in the K5, K5L, K5L-Luc cell lines was measured (A,B,C respectively). The expression levels were all compared to that of the mRNA of the K5 cells, the poor metastatic parental cell lines. Each point shows the mean expression variations (±S.D.) measured using mRNAs prepared from three experiments carried out independently. The mean fold change in expression of the target gene in each cell line versus K5 cells was calculated using the method 2^−ΔΔC^_T_.

As *in vitro* growth properties of K5 and K5L cells did not differ, we wondered whether *in vivo* properties were associated to the acquisition of specific expression profiles. To characterize these cell lines, we performed expression profiling of these and other clones derived from the spontaneous OSA, arose in the distal femur of a Balb/c mouse [15] and which were reported to be tumorigenic but not invasive *in vitro* and metastatic *in vivo*. In the same assay, we tested other clones displaying invasiveness *in vitro* and tumorigenic and metastatic phenotype *in vivo* [16]. This analysis (not shown) allowed us to identify three genes, which had the highest differential expression in the *in vivo* metastatic K5L cells (*enpp4*, *prkcd* and *pfn2*). By means of qPCR, we demonstrated the significantly increased expression of *enpp4* (Fig. 2A, p<0.001) and *prkcd* (Fig. 2B, p<0.04) and the reduced expression of *pfn2* (Fig. 2C, p<0.001) in metastatic cells. Interestingly, expression of these three genes has been already associated to the metastatic phenotype of human cancer cells [21]–[25].

### Imaging tumorigenesis and metastasis *in vivo* using IVIS

Although the metastasis-derived cells showed slight increase tumorigenicity, *in vitro* growth, motility and invasion assays showed superimposable phenotypes. We found differential expression of three metastasis associated genes. Thus, we wondered whether the metastatic phenotype of the metastasis-derived K5L cells was maintained by cells propagated in culture.

**Figure 3:**
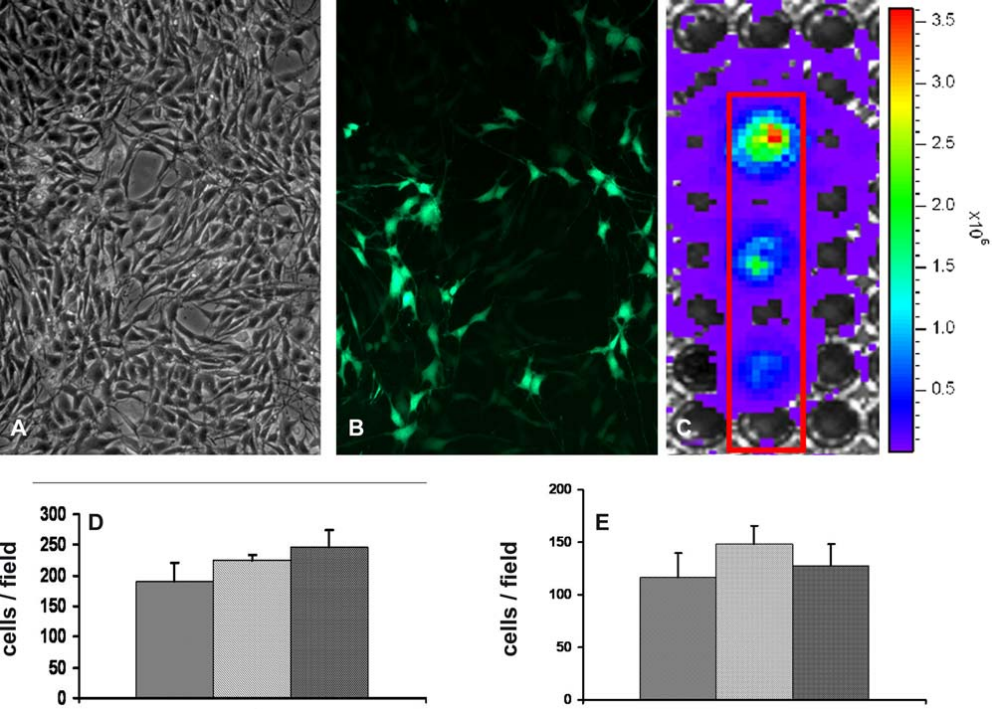
K5L cells infected by lentivirus. K5L culture cells (A) infected by lentivirus, named K5L-Luc, constitutively express both GFP (B) and Luciferase (C) protein. Notably over 40% of the cells plated were positive for GFP (B). Cells were plated in a 96 multiwell plate in various dilutions and they showed a detectable luciferase signal using IVIS. From the top, wells were plated with 1,5×10^5^, 1×10^5^ e 5×10^4^ cells. Signal intensity was roughly proportional to cell number (C). Transwell assays to compare the *in vitro* K5, K5L, K5L-Luc cells migratory and invasive ability were performed in starving condition. Cells migrated through the porous filter were counted after 16 h incubation (D). Cells migrated through the artificial basement membrane (Matrigel®) were counted after 24 h incubation (E). In both panels Y-axis shows average migrated cell number (±S.D.) of triplicate wells in a representative experiment.

The metastatic K5L cells were engineered to stably and constitutively express both the Green Fluorescent Protein (GFP) and Luciferase by lentiviral-mediated gene transfer. By microscopy GFP fluorescence allowed to verify that approximately 40% of the cells presented viral integration. By seeding increasing amounts of the bulk-infected cells in a multiwell plate and subjecting it to IVIS, we determined that a Luciferase signal was detectable starting from the highest cell dilution (50,000 cells/well) and that signal intensity was roughly proportional to cell number (Fig. 3 A–B–C).

In order to rule out possible modifications of the neoplastic phenotype induced by lentiviral infection, we verified that *in vitro* K5L-Luc cells had migratory and invasive ability comparable to K5 and the K5L, without showing any statistically significant differences (Fig. 3 D–E). Moreover, we found that expression profiles of K5L-Luc cells were not modified, as also demonstrated by selected transcript validation (Fig. 2 A–B–C).

For the *in vivo* experiments, we selected as marker the Luciferase reporter protein, since bioluminescent Luciferase imaging proved to be more sensitive than fluorescent GFP imaging. Furthermore, the GFP signal is not sufficient to overcome tissue scattering from more than a few millimeters' depth [26], making it unsuitable to monitor metastatic localization.

**Figure 4:**
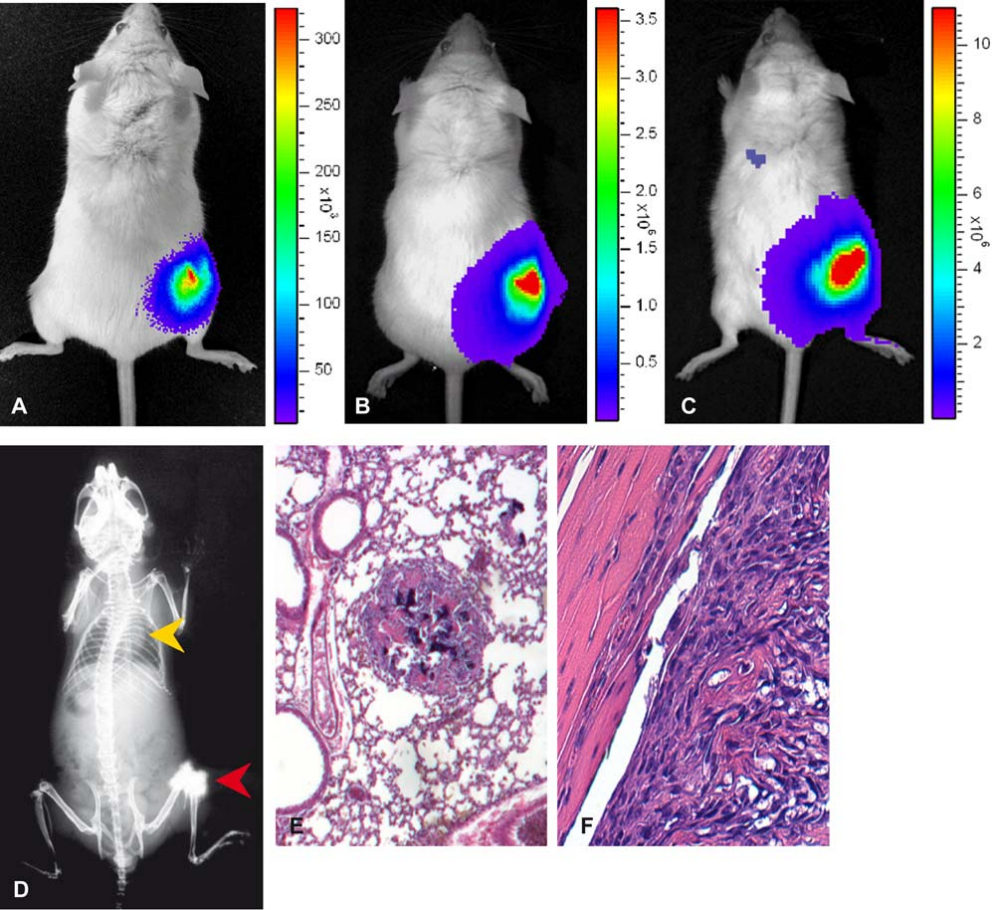
K5L-Luc mouse *in vivo* imaging and histologic evaluation of the primary tumors and pulmonary metastases. Fifteen minutes after injection of 1×10^6^ K5L-Luc cells the signal from labeled cells was localized in the injection site of the right knee of the mouse (A). On day 21 from inoculation, tumor growth at the injection site (right knee) was macroscopically detectable and documented using IVIS. The only source of emission is circumscribed to the injection site (B). Thirty-eight days after K5L-Luc cells injection, a first luminous signal was evident at pulmonary level (C). X-ray analysis (D) revealed an aggressive destructive growth pattern toward knee region with periosteal new bone formation at the site of injection (red arrow), but did not show sign of metastatic spread to lung (yellow arrow).At 45 days histologic analysis of the K5L-Luc orthotopic primary tumor section (F) revealed densely packed malignant mesenchymal cells consistent with osteoid formation and bone trabeculae, including invasion of normal bone (Magnification 10×). Non-sequential serial sections were taken from lungs of the injected K5L-Luc animals and section through a visible pulmonary metastasis (E) revealed malignant mesenchymal cells proliferating in the lung parenchyma. Staining with eosin hematoxylin was performed (Magnification 40×).

We determined the ability of the K5L and K5L-Luc cells to develop spontaneous lung metastases in Balb/c mice. Experimentally, 10^6^ cells were injected into the distal femur of Balb/c mice, and then we checked the K5L-Luc cell localization immediately after injection using IVIS (Fig 4A). Subsequently, we weekly checked the injected animals for Luc bioluminescence by IVIS to monitor tumor growth and to visualize the presence of distant metastases in the animals (Fig. 4B). At 21 days after injection, primary tumors were macroscopically detectable in all mice of both groups. At 38 days, we observed by IVIS the first sign of metastasis in one animal (Fig. 4C). Numerous metastases could be detected by IVIS during the subsequent week in every animal of this group. At 45 days after tumor cells injection, the animals of both groups were sacrificed for ethical reasons since their condition worsened, and they developed serious dyspnea. It should be noted that the X-ray analysis to detect metastases which were visualized by IVIS. In fact, 38 days after the injection, X-rays of animals did not show sign of lung metastatic spread (Fig. 4D), while the new bone formation caused by the primary tumor was clinically and radiographically evident (Table 1: OSA cell lines tumorigenesis and metastatic ability).

**Table 1:** OSA cell lines tumorigenesis and metastatic ability

Cell line	Animals with primary tumor at 21 days (%)	Tumor-bearing animals with lung mts ˆ at 45 days (%)
**K5**	60 _6/10_ *	30 _2/6_ °
**K5L**	90 _9/10_ *	100 _9/9_ °
**K5L-Luc**	100 _10/10_ *	100 _10/10_ °

ˆ : metastases

* : number of animals with primary tumor/number of injected animals

° : number of animals with lung metastases/number of animals with primary tumor

The histology of the primary tumors showed solid growth with high proliferation index, extensive osteoid matrix and bone formation, characterized by highly cellular masses of densely packed large mesenchymal cells (Fig 4F). The K5L-Luc cells permeated the bone trabeculae to implement bone destruction. Histological analysis of the pulmonary tissue showed tumor foci characterized by high-grade proliferating tumor cells. These metastatic cells were malignant mesenchymal cells similar to the primary tumor cells (Fig 4E).

**Figure 5:**
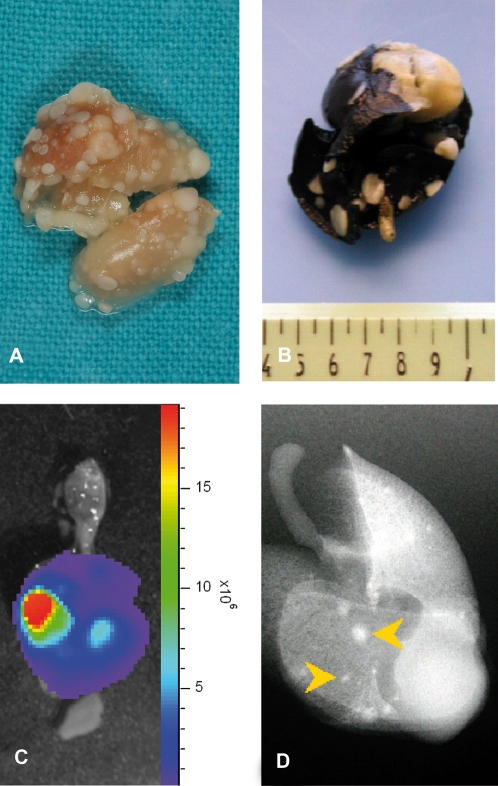
Lung metastases' macroscopic analysis. At 45 days after tumor cells injection, the animals injected with K5L and K5L-Luc cells were sacrificed. Macroscopic surface metastases were observed in all lungs (A). The lungs of the mice injected with K5L cells were removed and stained with India ink (B).Using IVIS we analyzed lungs of animals injected with K5L-Luc cells (C). When the isolated lungs were exposed to X-ray, the latter highlighted calcified metastasis (D, yellow arrow).

The lungs of the control mice injected with K5L cells were removed and were stained with India ink to confirm the presence of metastases macroscopically. Macroscopic surface metastases were observed in all lungs (Fig 5A–B). Moreover, all mice were evaluated for the presence of metastatic localization in other organs. Macroscopically, we did not observe nodules on any other organs. Using IVIS we analyzed all animals injected with K5L-Luc cells and we did not visualize metastases in liver or in other abdominal organs.

The overall expression profile of the Luciferase engineered K5-Luc cells was superimposable to that of the parental metastasis-derived K5L cells (data not shown). Indeed the differential expression of the three metastatic related genes was similar (Fig. 2).

Altogether these data show that metastatic ability is a property stably retained in this model, even after propagation in culture and gene marking.

## Discussion

Before 1975, the treatment of appendicular OSA mainly consisted of amputation without effective chemotherapy. The introduction of adjuvant and neo-adjuvant chemotherapy led to a significant improvement in prognosis [27]. Moreover, changes in the surgical treatment towards limb sparing techniques instead of amputation, increased quality of life of OSA patients [28].

At the time of diagnosis, significant prognostic factors are the presence of metastases, followed by tumor volume and percentage of tumor necrosis at primary site after neo-adjuvant chemotherapy [29]. Despite the successful surgical treatment of the primary site, the presence of micrometastases, resistant to chemotherapeutic regimens, is responsible for the failure of the cure. Therefore, the study of lung metastases is needed to improve treatment outcomes and to identify patients with the highest risk for metastatic spread.

Metastatic models of tumorigenesis in experimental animals are rare even though they are fundamental to understand the cancer invasive behavior. Studies of human OSA were conducted in nude mice or otherwise immuno-compromised mice [7], [30] and in some of these cases OSA was induced by injecting human tumor cells either intravenously or subcutaneously [20]. These mice models might not be clinically relevant because OSA cells are not spontaneously arisen and do not grow in the proper site. Moreover, tumor progression and metastases development do not mimic the human clinical course of OSA and lack immunological response. Otherwise, the heterologous models might bias the biology of the disease involving response to histocompatibility antigens and/or other species specific moieties.

We focused on a mouse spontaneous OSA, which is characterized by spontaneous pulmonary metastases after orthotopic implant of tumor cells [15], [31]. In this orthotopic OSA model tumor progression and metastasis development follow the human clinical scenario. Thus we used it to study the migratory and invasive properties of a new OSA cell line, able to form osteogenic tumors and metastases in syngeneic Balb/c mice. We show that the metastasis derived cells are slightly more tumorigenic but strongly more metastatic than the parental ones. Interestingly, gene expression analysis showed that three genes are differentially expressed in the metastatic cells (*prkcd*, *pfn2*, *enpp4*). These genes were previously correlated to cancer cell invasion and metastasis. In particular, *prkcd* triggers cell migration and confers metastatic potential to a variety of normal and tumor cells, such as breast [21], [22] and gastric cancer cells [23]. In agreement with our results, a dramatic down regulation in the expression of *pfn* isoforms has been found in invasive breast cancer cells compared with its non tumorigenic counterparts using gene expression profiling [30]. Furthermore, overexpression of *pfn* in breast cancer cells resulted in changes in morphology and cytoskeletal organization and caused suppression of tumorigenicity in a nude mouse model [30]. The aberrant expression of *enpp* family has been involved in cell motility and migration, angiogenesis, tumor cell invasion and bone mineralization dysfunction but the underlying mechanisms largely remain to be determined [31].

The K5L cell line was transduced to express luciferase using Lentivirus vector, which integrate efficiently into the genome of the target cells. It is known that Lentivirus vector do not transfer any viral gene and they can be used for stable *in vivo* gene delivery in non-dividing cells without affecting morphology, marker proteins and characteristic behavior specific of the parental cells [32]. As expected, after lentiviral infection we did not observe changes in K5L-Luc cells biological properties compared to parental K5L cells.

The K5L-Luc cells formed tumors locally in the injection site and produced numerous spontaneous metastases. Interestingly, *in vivo* assays and expression profiles showed that the metastatic phenotype is a stable property acquired by cells growing *in vivo*, stably maintained after *in vitro* propagation and not modified by Lentiviral trasduction.

Many strategies to visualize tumor cells in animal models have been reported [7], [33], [34] but to our knowledge, this is the first report of a murine syngeneic Balb/c, spontaneous OSA model in which OSA cells are detectable in real-time by *in vivo* imaging. In fact, this approach, based on a spatio-temporal imaging analysis of the metastatic process, does not require the sacrifice of mice at selected time-points to evaluate tumor and metastasis growth. The optical detection of luminescent tumor cells *in vivo* represents a powerful tool to follow the tumor progression in laboratory animals. It is a reliable system to evaluate anti-osteosarcoma strategies, especially against post-surgical micrometastatic disease. The IVIS use for non-invasive quantification of reporter gene expression (luciferase) from live cells as well as from live animals is helpful for fast screening at relatively low cost.

Finally, in human and veterinary preclinical study, considering the high incidence of naturally occurring OSA in dogs, this mouse model could be used as a tool, to obtain a proof of concept of the efficacy of novel therapeutic approaches.
